# Development and Testing of a Clinical Practice Framework for Pharmacists to Assess Patients’ Travel-Related Risks: The 5W Approach to Travel Risk Identification

**DOI:** 10.3390/pharmacy7040159

**Published:** 2019-11-26

**Authors:** Heidi V.J. Fernandes, Sherilyn K.D. Houle

**Affiliations:** School of Pharmacy, University of Waterloo; Waterloo, ON N2L 3G1, Canada; heidi.fernandes@uwaterloo.ca

**Keywords:** travel medicine, pharmacists, travel, feasibility studies, vaccination, risk assessment, validation studies

## Abstract

**Objective**: To assist with identifying patients who may be managed by pharmacists without additional travel medicine training, versus those who may benefit from referral, we developed and validated a clinical practice framework. This framework was then piloted in eight pharmacies in Ontario, Canada, from March to August 2019. **Methods**: A panel of experts, comprised of physicians and pharmacists from Ontario, Canada, holding a Certificate in Travel Health^TM^ from the International Society of Travel Medicine was recruited. This panel participated electronically in the development of the framework in three stages: (1) Sharing their current approach when performing information gathering and assessing risk in a traveling patient; (2) judging of items collated from all panellists on the basis of how essential they are to a risk assessment; and (3) validation of items deemed essential by the panel using the Item and Average Content Validity Index. The framework was then released to community pharmacies, where pharmacists that self-identified as beginners to travel medicine completed pre- and post-test phase surveys to determine the utility of the framework. **Key Findings**: A total of 64 items for consideration were deemed essential enough to proceed to content validation, organized into 5 ‘W’ domains: Who, What, Where, When, and Why. Each item was ranked by the experts according to its relevancy, resulting in an Average-Content Validity Index of 0.91. The resulting framework was titled “The 5W Approach to Travel Risk Identification.” This clinical practice framework is the first published assessment tool for travel medicine tailored for pharmacy’s scope of practice that has been content validated. Pharmacists reported that the framework is simple to use and provides structure for interactions with travelling patients. However, it may not be as beneficial for those with a higher level of travel medicine expertise than the average pharmacist. **Conclusion**: The 5W Approach tool allows pharmacists inexperienced in travel medicine to collect information when required to use their professional judgement when assessing traveling patients as either high-risk (requiring a referral to a travel medicine specialist) or low-risk. With the aim of supporting pharmacists to be more confident in caring for traveling patients and increasing their involvement in travel medicine, future research will test this framework for feasibility in Canadian community pharmacy practice.

## 1. Introduction

In December 2016, the government in Ontario, Canada expanded the scope of pharmacists’ immunization administration authority to include 13 vaccine-preventable diseases in addition to the influenza vaccine [1]. Although expansions in scope are generally well-received, legislative changes alone do not directly result in practice changes. As observed with previous expansions to scope (for example, adapting and renewing prescriptions or conducting medication reviews), pharmacists’ uptake of new roles and responsibilities can be a gradual process, and there may be hesitation to implement it into practice [2,3,4,5]. 

A survey of community pharmacists, approximately two years following scope expansion in Ontario, found that the initial uptake of this scope expansion was slow, with 94% of respondents reporting that they administered fewer than 10 of the new vaccinations added to their scope per month. Of note, these also included non-travel vaccinations, such as herpes zoster and human papillomavirus vaccinations, which represented the second- and fifth-most frequently administered vaccines, respectively [6]. When asked about the new vaccinations, pharmacists cited varying levels of confidence with administering or recommending vaccinations for travel. This was attributed to lower familiarity with the vaccines and a perceived lack of clinical knowledge in travel medicine [6]. 

The results of the aforementioned survey align with previous studies regarding pharmacists that are beginners to the field of travel medicine. When reviewing the literature surrounding pharmacists’ care in travel medicine two themes emerge:

1. Given extensive postgraduate training and experience in practice, pharmacists can positively impact health outcomes among travellers [7,8,9,10,11].
2. Given the entry-level competencies required for pharmacists to practice, and lack of travel medicine training in pharmacy school curricula, most pharmacists without additional training or experience in travel medicine feel inadequately prepared to care for travelling patients. Further education and training regarding travel medicine for pharmacists are also often discussed as strategies to be explored by the studies with this theme [12,13,14].

Travel medicine expertise is often defined as an individual holding the International Society of Travel Medicine’s (ISTM) Certificate in Travel Health^TM^ (CTH^®^) and/or those who have completed a post-baccalaureate Doctor of Pharmacy (PharmD) with or without a hospital-based or primary care residency. These pharmacists exhibit confidence in their care, demonstrated through the creation of their own travel health clinics, perceived self-competence, and strong patient outcomes. Pharmacists with travel medicine expertise have been found to consistently make evidence-based recommendations concordant with guidelines and their patients report a high level of satisfaction, including acceptance of recommendations and a sense of preparedness to manage health conditions that arise while travelling [7,8,9,10,11]. However, the literature indicates deficiencies when evaluating care provided by pharmacists without additional travel medicine training. Although pharmacists are interested in travel medicine, they report not feeling adequately prepared for it, which has resulted in lack of patient education regarding oral typhoid vaccination, incomplete and/or incorrect travel advice, and recommendations regarding rabies pre- and post-exposure prophylaxis discordant with guidelines [12,13,14].

Related to pharmacists’ need for further training or education in travel medicine, the complexity of travel medicine (for example, regional differences in disease epidemiology, outbreaks, and changes in resistance patterns for infectious diseases) and need for individualized care is noted by pharmacists. Many preliminary studies have identified this barrier and suggest that a gap in training in pharmacy school curricula is a contributor [15,16,17,18]. Pharmacy schools across the US and Canada do not have robust travel medicine competencies built into their core curricula, apart from immunization training, which tends to focus on influenza and other routine vaccinations [15,16,17,18]. This lack of exposure during pharmacists’ training years as students likely impacts the provision of these services upon licensure. Further investigations into the scope of this problem need to be completed in order to make a definitive conclusion on the extent of this as a contributor and strategies to best address it.

Given the historical pattern of uptake of expansions to pharmacy practice, similar challenges are anticipated regarding travel medicine activities, which may be amplified by additional practical factors such as lack of confidence with therapeutic knowledge in the area, lack of direction and support for the new service, and challenges with integrating the new service into the pharmacist’s existing workflow. Previous studies surveying pharmacists’ opinions have mentioned that an educational aid or practice tool may be a valuable facilitator to increase the uptake of travel medicine services [6,15]. 

To address these factors, we created and content-validated a questioning framework that pharmacists can use to triage risk factors among travelling patients. Pharmacists currently utilize various frameworks to guide patient assessments across a number of therapeutic areas. These frameworks are especially helpful to those new to the areas, such as students and new practitioners; however, even experienced clinicians continue to refer to frameworks to ensure a consistent approach to their patient assessments and documentation. For example, assessments related to patient self-care of common ailments often follow the “SCHOLAR” (Symptoms, Characteristics, History, Onset, Location, Aggravating factors, Remitting factors) and “MACS” (Medications, Allergies, Conditions, Social history) mnemonics [19]. Similarly, the “OPQRST” (Onset, Palliation and Provocation, Quality and Quantity, Region and Radiation, Signs and Symptoms, Temporal relationship) mnemonic is valuable to the assessment of pain [20]. These frameworks provide health professionals with a structure to perform these assessments upon, adding to their confidence that their assessment will not miss any important elements that may affect their clinical decision-making.

The current literature contains no published frameworks to assist pharmacists in the area of travel medicine. While a number of clinical practice guidelines and publications, such as the Centers for Disease Control and Prevention’s (CDC) Yellow Book 2020: Health Information for International Travel, exist, little guidance is provided on how to interpret and implement this information into practice [21,22,23]. This results in a wide variety of treatment experiences for the traveling patient and inconsistencies in the assessment of a traveling patient’s healthcare needs. Of the resources available, none are tailored for applicability to the pharmacy profession (e.g., different practice sites, scope of practice, approach to patient assessments). 

The objective of this study was to create an expert-informed validated clinical practice framework that pharmacists can use for risk assessment of traveling patients. The following article details the development and preliminary testing of the framework in community pharmacies and the impact this framework had on pharmacy practice in Ontario, Canada.

## 2. Materials and Methods 

**Figure 1:**
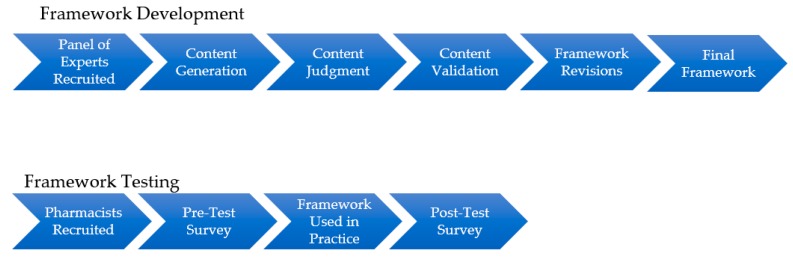
Methodological process.

Ethics approval for both the development and testing phases of the study was received from the University of Waterloo Research Ethics Committee (ORE #40021). Figure 1 describes the overall methodological process of this study.

### 2.1. Framework Development

The framework was developed in four stages: content generation, content judgement, validation, and final framework production. A panel of experts known to the authors was recruited to complete the first three stages. All interaction with the panel was done electronically through email communication. 

The criterion used to define our subject matter experts was a healthcare professional that had obtained the International Society of Travel Medicine’s Certificate in Travel Health^TM^ (CTH^®^). The CTH^®^ is an internationally-recognized designation, which indicates that the person understands a wide body of knowledge related to travel medicine [24]. Currently there is no consensus on the number of subject matter experts recommended to develop or review an instrument [25]. Although the more experts included decreases the probability of agreement due to chance and can better inform the framework’s development, the maximum number is often up to 10 experts [25,26]. In order to eliminate split decisions, while still gathering sufficient input, a panel of 9 experts was recruited. 

#### 2.1.1. Content Generation

An open-ended question was posted to the panellists to gather a list of items to consider for the framework: “What information do you gather to ascertain a traveling patient’s risk and what questions do you ask to obtain that information?” All items collected in this stage were collated, organized into broad domains, and considered in the following content judgement stage.

#### 2.1.2. Content Judgement

Each item identified in stage 1 was included in a web survey, administered using Qualtrics^TM^ software (Qualtrics, Provo, UT, USA), which asked panellists to categorize each item as one of: essential; useful, but not essential; or not necessary. Only those items that were categorized as essential by more than half of the panellists (n ≥ 5) moved on to the content validation stage. Experts were also given the option at the end of the survey to provide any comments, such as the addition, deletion, or re-wording of any item(s), which would be considered in subsequent stages.

#### 2.1.3. Content Validation

The quantitative index used to measure content validity for the framework was the Content Validity Index (CVI). The CVI involves the panel of experts rating each item based on content relevance or representativeness for an instrument and is considered the most widely utilized method of quantifying content validity [25]. The panel was asked to rank the relevancy of each item that can be used in determining whether the traveller is a low- or high-risk patient. This was administered via another web survey using Qualtrics^TM^ software (Qualtrics, Provo, UT, USA). The ranking was based on a 4-point Likert scale (1: not relevant; 2: somewhat relevant; 3: quite relevant; 4: highly relevant). A 4-point scale was selected over a 3- or 5-point rating scale because it does not contain a midpoint rating, forcing the expert to make a choice as opposed to being neutral or unsure, and also allows provides adequate information to calculate a CVI [25,27].

To quantify the framework’s validity, each item’s content validity index (I-CVI) was calculated, in addition to the overall Average Content Validity Index (Ave-CVI). The I-CVI was calculated “by counting the number of experts who rated the item as a 3 or 4 and dividing that number by the total number of experts, that is, the proportion of agreement about the content validity of an item” [28]. There are many ways to calculate an instrument’s Ave-CVI (e.g., the proportion of items rated relevant across experts can be averaged, the I-CVIs can be summed and divided by the number of items, or the total of number of ratings as a 3 or 4 can be counted and divided by the total number of ratings); for this study, all I-CVIs were averaged to calculate the Ave-CVI. It is important to note that all three methods for calculating the Ave-CVI will yield the same value, but it has been suggested that averaging the I-CVIs is “more related to the quality of the items rather than the performance of experts” [29]. As a valid framework is defined as having an Ave-CVI ≥ 0.90, if not achieved in the first round of surveys, items will be revised and recirculated to the panel until this value is obtained.

#### 2.1.4. Construction of The Framework

Construction of the framework involved the organization of each included item into domains using a checklist format to facilitate ease of use in practice. Following content validation, a preliminary framework was made. To ensure understandability and face validity, 3 Canadian-licensed pharmacists that had been practicing for less than 5 years and had no formal training or self-identified expertise in travel medicine were asked to review the framework for clarity and provide feedback, as they represent a potential user group of the framework. The framework was subsequently revised until each of the pharmacists expressed satisfaction with it.

### 2.2. Framework Testing

#### 2.2.1. Study Design and Recruitment

To obtain an initial evaluation of the framework, we used a pre-and post-test study design with the availability of the framework being the intervention. Pharmacist participants were recruited using personal contacts of the researchers, including previous participation in travel medicine studies. Recruitment of the participants was ongoing from January to April 2019. The inclusion criteria for the pharmacists was:

1. Current practice is in a community pharmacy.
2. Part A (able to provide direct patient care) licensure through the Ontario College of Pharmacists (OCP) or 4th year entry-to-practice PharmD student currently on clinical practice rotation.
3. Does not currently hold CTH^®^ designation from ISTM. This exclusion was applied as it is a global indicator that the individual has an advanced level of travel medicine knowledge [24], whereas this framework was developed specifically for pharmacists without experience or expertise in travel medicine.

#### 2.2.2. Data Collection

The testing took place from March to August 2019 at community pharmacies across Ontario, Canada. During this study period, pharmacists were instructed to “Please utilize the framework in your practice, as you deem fit, to triage patients as either high or low risk travellers.” If the framework was used, the pharmacists were asked to record metrics on the back of the framework. These metrics included the date used, estimated triage time (minutes), whether the patient was referred or not and the reasoning behind the decision made. These metrics were then faxed to the researchers at the end of each month. 

At the time of enrolment, participants were asked to complete a survey to gather baseline information on their demographics, practice-related characteristics, and current practices regarding travel medicine (Appendix A). Pharmacists were also asked to complete an online survey in September 2019 once the study period had concluded. This survey gathered feedback on the framework’s feasibility and impact on pharmacy practice (Appendix A). Feedback was gathered using open-ended questions that allowed participants to describe the main advantages and disadvantages of the framework, as well as provide any suggestions for improvement and detail how pharmacists saw the framework being incorporated into their pharmacy workflow.

All surveys were administered using Qualtrics^TM^ software (Qualtrics, Provo, UT) with questions collecting both quantitative and qualitative data, using free-text answer formats. Descriptive statistics were performed using Microsoft Excel for Windows 10, Version 1902 (Redmond, WA, USA).

## 3. Results

### 3.1. Framework Development

As pharmacists are the intended primary audience for the tool, seven of the nine experts recruited were pharmacists, and the remaining two were family physicians (Table 1). 

Panellists submitted their responses online for content generation (stage 1) in a variety of formats, including detailing their thought process with a traveller, or submitting resources and/or questionnaires used in their practices. A total of 114 unique items were identified in stage 1, which were organized into 6 domains of information gathering, as indicated in Table 2:

Rankings on the essentialness of the 114 items in stage 2 are provided in Appendix B. The response rates of panellists for stages 1 and 3 of the study were each 100%. However, the response rate for stage 2 was 78% (n = 7) due to the unavailability of two panellists during the data collection period. Despite fewer panellists participating in stage 2, the decision was made to still require 5 or more of them to deem an item to be essential for it to be included in the content validation stage.

In total, 64 items were categorized as essential and moved on to content validation. At this point, the “How” domain was removed completely from the final framework as none of its items were ranked essential, leaving the 5 ‘W’ domains of *who*, *what*, *where*, *when*, and *why*. A full breakdown of how those 64 items were ranked according to relevancy, including their I-CVI, can be found in Table 3. The Ave-CVI across all items was calculated to be 0.91. Upon re-consideration, 2 items regarding dining were switched from the “Where?” to the “What?” domain for appropriateness. Additionally, further information on the definition of immunocompromised status and a list of countries that could be considered high-risk was added following framework review by practicing pharmacists.

**Figure 2:**
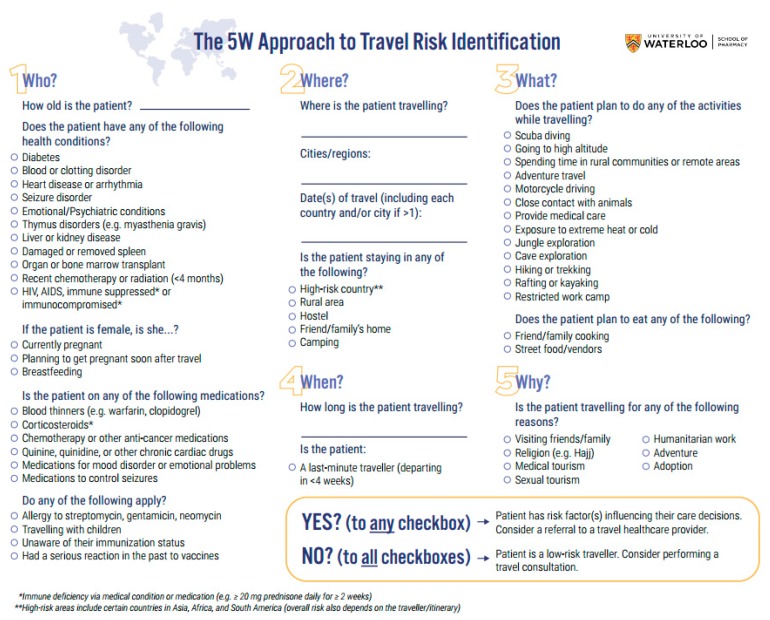
Final version of The 5W Approach to Travel Risk Identification framework.

The final version of the framework (Figure 2) is a concise one-page tool to identify risk factors in traveling patients. While pharmacists with any level of travel medicine experience are welcome to use the framework, it is primarily meant for those with minimal experience. As the intended user group consists of community pharmacists new to the field of travel medicine, the items are primarily posed in Yes/No question format, where a positive response to any item may indicate a need for referral to an experienced travel medicine healthcare professional. Those items that are posed as open-ended questions allow for the pharmacist to use their judgement on determining whether the patient’s response is a criterion for referral. If these answers are deemed low-risk and the patient has answered “no” to all the other questions, the patient can be classified as a low-risk traveller that could likely have a travel consultation done by that pharmacist.

### 3.2. Framework Testing

Of the 19 respondents that reviewed the invitation letter and expressed interest in testing the framework, nine were excluded for failing to provide consent to participate and two participants were excluded due to failure to complete the pre-study survey. One participant failed to complete the post-study survey. The demographics of the eight respondents completing the study is provided in Table 4. Half of the respondents indicated practicing in a chain pharmacy and in the capacity of a staff pharmacist. Most (n = 5) practiced in South West Ontario, consistent with the greater population density in this region of the province [30]. Most pharmacists (n = 5) had 11 or more years of experience, all were authorized to administer injections, and most had a Bachelor’s degree as their highest level of pharmacy education. Additional training was completed by one participant through the American Pharmacists Association’s Pharmacy-Based Travel Health Services continuing education program [31].

All pharmacists that reported some experience with the additional vaccines added to the scope of practice to varying degrees. However, their approach when interacting with a travelling patient varied. Prior to this study, when a patient presented to the pharmacy inquiring on precautions they need for an upcoming destination, participants reported they may provide information on general precautions (n = 7), perform a complete consultation for less complex patients (e.g., all-inclusive resort in the Caribbean, cruise) and refer all others (n = 6), refer all patients to a travel clinic or to their physician (n = 3), refer patients to online or paper resources with more information (n = 3), or other (n = 1) which was described as “review complex patients for risks associated [with pre-existing medical conditions] and notify GP (e.g., anticoagulation).”

#### 3.2.1. Attitudes and Beliefs Towards Travel Medicine

All pharmacists expressed a high degree of willingness to incorporate travel medicine into their practices. The primary motivators included travel medicine questions being increasingly frequent from their patient populations and pharmacists’ self-interest in travel medicine. As Pharmacist 3 explained, “travel is more and more common and with pharmacists able to give some vaccinations it should be an expectation of patients to get help in any retail pharmacy.” Pharmacist 6 commented that “[travel medicine] is a relevant and essential part of patient care that most times does not require a lot of effort.” The primary barriers cited preventing the participants from starting travel medicine services includes lack of knowledge regarding travel medicine, lack of time, and lack of prescribing authority. Regarding knowledge, Pharmacist 5 stated “pharmacists underestimate the complexity of knowledge required for travel medicine practice. Pharmacists do not have enough knowledge nor training on vaccinology or disease knowledge required for a travel consult. If a consult is not done properly, we are doing patients a disservice.” When referring to the inability to prescribe, Pharmacist 2 noted that “some physicians will accept recommendations from the pharmacist and send an Rx, but others refer everyone to a travel clinic.” It is also important to note that participants appeared to use the terms “counselling” and “travel consultation” interchangeably through the survey questionnaires. Its implications are detailed in the discussion section.

#### 3.2.2. Framework Feasibility

The framework was only utilized in March and April of the 6th month study period, totalling three interactions. The results of the pharmacists’ interactions are recorded in Table 5.

Despite the framework not being used by all pharmacists during the study period, feedback was sought from all participants in the post-test survey (n = 7). Overall, it was viewed as a helpful tool that can guide pharmacists with questions and identify complex patients that may need referral beyond a pharmacist’s scope. Benefits included being simple to use and asking the important questions for assessing a travelling patient while providing a structure for pharmacists to follow. As Pharmacist 6 commented, “[you] can follow an algorithm to assist in guiding decisions, especially if encountering a complex situation.” Participants did note that the framework contained a lot of text, which resulted in a time investment required to orient oneself to the intended flow. Time investment in completing the framework could also be a limitation if it is identified near the end of the framework that the patient has a complicating factor warranting referral. As Pharmacist 4 explained, “[the] patient might be upset that after all the questions and discussion, they still have to go to the travel clinic.” While it was generally noted as a great tool for most pharmacists, it may be less useful for pharmacists with more education in travel health, who may have their own preferred format. 

## 4. Discussion

### 4.1. Framework Development

Following expert-informed content generation, judgement, and validation, we produced a succinct clinical practice framework intended for community pharmacists to triage the risk profiles of traveling patients. It is the first tool of its kind targeted to pharmacists to identify patients who may be safely assessed in a community pharmacy by a pharmacist with limited travel medicine training or experience, versus those who would benefit from referral to another clinician. The 64 items included largely align with pre-travel risk assessment recommendations included in the CDC’s Yellow Book and other references [21,22,23,32] and are grouped into five broader domains (the 5 Ws of Who, What, Where, When, and Why) for ease of understanding and use.

Successful content validation, defined as Ave-CVI ≥ 0.90 [28], was achieved after only one round of content validation. Additionally, it should be noted that the expert panellists practiced in different locations across Canada, reflecting perspectives from different provinces where scope of practice can vary. Depending on the province, pharmacists’ scope of practice can range from independently prescribing for all conditions related to travel medicine, through prescribing within certain legislative conditions or with a medical directive, to only being able to immunize against travel-related vaccine preventable diseases without prescribing authority [33]. The inclusion of a broad sample of pharmacists practicing under different scopes in the expert panel is expected to enhance the framework’s applicability across jurisdictions.

However, our work is not without limitations. The expert panel’s degree of input was limited, as all feedback was performed via online surveys consisting largely of multiple-choice questions. Open-ended feedback or rationale for selections was not sought, and panellists did not have the opportunity to discuss their selections with the other panellists. For example, while acceptable I-CVI values are those above or equal to 0.78 [28], 6 of the 64 items included in stage 3 did not meet this standard. Further revision of these items with the aim of improving their I-CVI was not performed. Additionally, the interpretation of each item’s relevancy was left solely to the discretion of the individual panellists. No further instruction was given or sought regarding the difference between 3 - Quite Relevant and 4 - Highly Relevant; however, this likely didn’t significantly affect the calculation of either the I-CVI or Ave-CVI as these depend on selecting either 3 - Quite Relevant or 4 - Highly Relevant. Another limitation was that two expert panellists were unavailable to provide input in the content judgement survey (stage 2), while all nine experts were able to participate in content generation (stage 1) and validation (stage 3). While the number of participants at each stage was sufficient [25,26,28], this discrepancy should be noted, as it represents slight differences in panel composition across each stage.

Pharmacists’ increasing involvement with clinical activities, particularly with travel medicine, is an emerging international trend, reflected by the creation of a Pharmacists Professional Interest Group within the International Society of Travel Medicine [34]. Previous travel medicine guidance documents on information gathering and risk assessment have either been targeted to the medical community or had limited accessibility to the broad pharmacist population (for example, embedded within continuing education modules, or internal questionnaires/frameworks created by pharmacy corporations). To our knowledge, this is the first framework for pharmacists to be published and, importantly, to also have its content validated. As a result, we are unable to compare our results to previous work.

### 4.2. Framework Testing

Overall, feedback on the framework from the pharmacist and final-year student participants was positive, with it reported to be an advantageous tool that is simple to use and can provide structure to guide pharmacists through travel-related interactions. However, it may not provide as much benefit for a pharmacist with above average travel medicine knowledge, which is to be expected as the intended audience was pharmacists new to travel medicine assessments.

The most significant limitation encountered in this feasibility study was the data collection period, as it ran from March to August 2019, which falls outside of the peak travel season for many Canadians who often opt to travel in the colder months of the year (November–April) [35]. Indeed, all uses of the framework occurred before May. In the monthly communications with the researchers, pharmacists reported throughout the study period that patients had not been coming in for travel advice, which hindered their ability to use the framework. The timing of the study is a potential reason for the low recruitment of pharmacists. The small sample size and minimal usage of the framework also affected the validity of the survey pharmacists were asked to complete once the data collection period concluded in August. Particularly, commentary provided on the framework from those who have not actually used it is not substantiated by experience with its use in practice. Finally, it should be noted that two of the three uses of the framework in practice was by pharmacy students. Despite not yet being licensed to practice independently, student participants were in their final year clinical practice rotations and can therefore be assumed to have similar knowledge and skills as a newly-licensed practitioner. One may argue that their level of exposure to formal travel medicine training may actually exceed that of many practicing pharmacists, as vaccines for travel is required learning in the second year of their program at the University of Waterloo, as is a two-hour lecture on travel medicine in their third year. However, their status as a student and need to potentially discuss assessments with their pharmacist preceptor may have contributed to the longer framework completion times observed among the trials conducted by the student participants.

Several studies have concluded that an educational aid or practice tool for pharmacists may serve as a facilitator to increase uptake of travel medicine services [6,15]; however, to date, no published studies have trialled the use of such a tool. As the first study to explore this type of work, a few implications on practice can be made. The low or non-existent use of the framework between May and August of the study period, due to patients not presenting to the pharmacist with travel-related inquiries, can impact the rate at which pharmacists are able to apply this expanded scope of practice in Ontario. As seen with our previous study on the uptake of immunization services, pharmacists’ confidence was directly related to the duration of scope availability and their frequency of exposure to it [6]. If there are limited opportunities for pharmacists to provide travel medicine services for half of the year due to low demand in the off-season for travel, it can be expected that an even slower rate of uptake may be observed relative to other clinical services provided year-round. For example, pharmacist prescribing for minor ailments has the potential for pharmacists to partake in that scope on a regular basis. That same regularity of exposure cannot necessarily be said for travel medicine. 

Another finding to investigate in future research is the quality of the care that pharmacists are providing for travelling patients. Despite participants self-identifying themselves as beginners in travel medicine, 75% (n = 6) of the pharmacists reported that their pharmacy offered pretravel consultations to their patients. Interestingly, only one participant reported charging a fee for this consultation. It would be highly unusual for pharmacies to not charge a fee for a comprehensive consultation that may take 30–60 min to complete [32]. This frequency is lower than that reported by respondents to our previous survey of Ontario pharmacists, which found that 35% of pharmacies offering travel consultations charged patients for this service [6]. As previously mentioned, the pharmacist participants appeared to use the terms “counselling” and “consultation” interchangeably, which may provide an explanation for these discrepant findings. The implications of this are two-fold:

1. Just because a pharmacy offers pretravel consultation services does not necessarily indicate that the pharmacists are actively performing them.
2. Pharmacists may have differing definitions of what they consider to be a pretravel consultation.

Variability in how pharmacists conduct pretravel consultations (e.g., via appointment or as an add-on to routine counselling on prescription or non-prescription drugs) can be a factor in this discrepancy as well. Variability in approach and comprehensiveness is not unique to travel medicine, as it was also observed following the introduction of the MedsCheck medication review program in Ontario [36]. For example, although approximately half of Ontarians with diabetes received an annual MedsCheck for Diabetes review, only 2.7–4.1% received a follow-up assessment, despite the use of potentially complex medications regimens for diabetes and comorbid conditions that warrant ongoing monitoring [36]. Although clinical effectiveness and high patient satisfaction have been observed from pretravel consultations performed by pharmacists with expertise in travel medicine [7,8,9,10,11], it remains to be determined if similar quality and consistency is observed when these services are offered by non-expert pharmacists. As one participant commented, “it would be a dis-service to the community if pharmacists are giving inadequate or bad advice. Pharmacy as a profession should not promote a service when members are not knowledgeable. Just because pharmacists are able to administer vaccines does not mean that pharmacists understand the disease the vaccine is there to protect.”

## 5. Conclusions

It has been established that the unique knowledge base required to practice in travel medicine contributes to lack of confidence among pharmacists in providing care for travellers. The 5W Approach to Travel Risk Identification provides a clinical practice framework for pharmacists that aims to address the challenges new practitioners in travel medicine may face when performing information gathering and general risk assessment of travellers. By being expert-informed and content-validated, this framework is expected to support pharmacists in the safe and effective identification of low-risk patients who may be manageable by a generalist practitioner versus those who may benefit from referral to another clinician with travel medicine expertise. Despite a small sample size of trials, the framework will be revisited as a potentially helpful tool that can guide pharmacists in the assessment of travelling patients. Further work needs to be performed to understand the full extent of the framework’s feasibility and impact on practice, as well as pharmacists’ understanding of what constitutes a pretravel consultation. Feasibility testing will be expanded to pharmacists across Canada, including different provincial scopes of practice, during peak travel season in the 2019–2020 period. 

## Figures and Tables

**Table 1:** Demographics of expert panellists.

Panellist	Profession	Gender	Year of Licensure	Year CTH^®^ Achieved	Practice Setting	Canadian Province of Practice
1	Physician	Female	1999	2007	Medical Clinic	Ontario
2	Physician	Male	2011	2013	Medical Clinic	Ontario
3	Pharmacist	Female	2009	2017	Community Pharmacy	British Columbia
4	Pharmacist	Female	1999	2011	Community Pharmacy	Nova Scotia
5	Pharmacist	Female	1994	2015	Community Pharmacy	Alberta
6	Pharmacist	Male	1993	2015	Consultant	Ontario
7	Pharmacist	Male	2012	2014	Community pharmacy	British Columbia
8	Pharmacist	Female	1999	2011	Travel Clinic	Alberta
9	Pharmacist	Female	2013	2015	Community Pharmacy	Ontario

**Table 2:** Domain identification and definition.

Domain	Definition
Who?	Patient specific-factors (e.g., medical conditions)
What?	Itinerary-specific factors (e.g., activities planned during travel)
When?	Timeframe of travel (departure date, duration at destination)
Where?	Country(ies) and region(s) visited, including order if more than one
Why?	Motivation for travel (e.g., visiting friends and relatives)
How?	Travel style and history (e.g., previous travel experience)

**Table 3:** Framework item and average content validation summary.

Item	Not Relevant (%, n)	Somewhat Relevant (%, n)	Quite Relevant (%, n)	Highly Relevant (%, n)	I-CVI
Domain: Who?					
Diabetes	0% (0)	11.1% (1)	33.3% (3)	55.6% (5)	0.89
Blood or clotting disorder	11.1% (1)	0% (0)	22.2% (2)	66.7% (6)	0.89
Heart disease or arrhythmia	0% (0)	11.1% (1)	33.3% (3)	55.6% (5)	0.89
Seizure disorder	0% (0)	11.1% (1)	33.3% (3)	55.6% (5)	0.89
Emotional/psychiatric condition(s)	0% (0)	11.1% (1)	33.3% (3)	55.6% (5)	0.89
Inflammatory bowel disease	0% (0)	33.3% (3)	22.2% (2)	44.4% (4)	0.67
Thymus disorders (e.g., myasthenia gravis)	0% (0)	11.1% (1)	11.1% (1)	77.8% (7)	0.89
Liver or kidney disease	0% (0)	11.1% (1)	33.3% (3)	55.6% (5)	0.89
Damaged or removed spleen	0% (0)	11.1% (1)	22.2% (2)	66.7% (6)	0.89
Organ or bone marrow transplant	11.1% (1)	0% (0)	0% (0)	88.9% (8)	0.89
Recent chemotherapy or radiation (<4 months)	11.1% (1)	0% (0)	0% (0)	88.9% (8)	0.89
HIV, AIDS, immune suppressed or immunocompromised	11.1% (1)	0% (0)	0% (0)	88.9% (8)	0.89
Currently pregnant	0% (0)	0% (0)	0% (0)	100% (9)	1
Planning to get pregnant soon after travel	0% (0)	0% (0)	44.4% (4)	55.6% (5)	1
Breastfeeding	0% (0)	11.1% (1)	55.6% (5)	33.3% (3)	0.89
Blood thinners (e.g., warfarin, clopidogrel)	0% (0)	0% (0)	44.4% (4)	55.6% (5)	1
Corticosteroids	0% (0)	0% (0)	11.1% (1)	88.9% (8)	1
Chemotherapy or other anti-cancer medications	0% (0)	0% (0)	0% (0)	100% (9)	1
Quinine, quinidine, or other cardiac drugs	0% (0)	0% (0)	44.4% (4)	55.6% (5)	1
Medications for mood disorder or emotional problems	0% (0)	11.1% (1)	44.4% (4)	44.4% (4)	0.89
Medications to control seizures	0% (0)	0% (0)	66.7% (6)	33.3% (3)	1
Age	0% (0)	0% (0)	55.6% (5)	44.4% (4)	1
Date of birth (for immunization purposes)	0% (0)	22.2% (2)	55.6% (5)	22.2% (2)	0.78
Allergy to streptomycin, gentamicin or neomycin etc.	0% (0)	11.1% (1)	44.4% (4)	44.4% (4)	0.89
Traveling with children	0% (0)	11.1% (1)	44.4% (4)	44.4% (4)	0.89
Awareness of immunization status	0% (0)	11.1% (1)	44.4% (4)	44.4% (4)	0.89
Serious reaction in the past with vaccines	0% (0)	0% (0)	22.2% (2)	77.8% (7)	1
**Domain: Where?**					
Country/Countries	0% (0)	0% (0)	11.1% (1)	88.9% (8)	1
Cities/Regions	0% (0)	0% (0)	22.2% (2)	77.8% (7)	1
Dates for travel for each country and/or city (if more than one)	0% (0)	0% (0)	77.8% (7)	22.2% (2)	1
Rural/urban areas	0% (0)	0% (0)	66.7% (6)	33.3% (3)	1
Hostels	0% (0)	11.1% (1)	55.6% (5)	33.3% (3)	0.89
Friend/family’s home	0% (0)	0% (0)	22.2% (2)	77.8% (7)	1
Camping	0% (0)	0% (0)	55.6% (5)	44.4% (4)	1
**Domain: When?**					
Departure/arrival dates	0% (0)	55.6% (5)	22.2% (2)	22.2% (2)	0.44
Last minute traveller (<4 weeks)	0% (0)	11.1% (1)	33.3% (3)	55.6% (5)	0.89
Length of stay	0% (0)	0% (0)	44.4% (4)	55.6% (5)	1
**Domain: Why?**					
Visiting friends/family	0% (0)	0% (0)	22.2% (2)	77.8% (7)	1
Athletic competition	0% (0)	44.44% (4)	33.33% (3)	11.11% (1)	0.44
Religion (e.g., Hajj)	0% (0)	0% (0)	22.2% (2)	77.8% (7)	1
Medical tourism	0% (0)	0% (0)	11.1% (1)	88.9% (8)	1
Sexual tourism	0% (0)	0% (0)	11.1% (1)	88.9% (8)	1
Humanitarian work	0% (0)	0% (0)	44.4% (4)	55.6% (5)	1
Adventure	0% (0)	11.1% (1)	44.4% (4)	44.4% (4)	0.89
Research/education	0% (0)	33.3% (3)	66.7% (6)	0% (0)	0.67
Adoption	0% (0)	0% (0)	44.4% (4)	55.6% (5)	1
**Domain: What?**					
Scuba diving	0% (0)	11.1% (1)	33.3% (3)	55.6% (5)	0.89
Going to high altitude	0% (0)	0% (0)	44.4% (4)	55.6% (5)	1
Safari	0% (0)	33.3% (3)	55.6% (5)	11.1% (1)	0.67
Spending time in rural communities or remote areas	0% (0)	11.1% (1)	44.4% (4)	44.4% (4)	0.89
Adventure travel	0% (0)	11.1% (1)	55.6% (5)	33.3% (3)	0.89
Close contact with animals	0% (0)	0% (0)	22.2% (2)	77.8% (7)	1
Providing medical care	0% (0)	0% (0)	11.1% (1)	88.9% (8)	1
Exposure to extreme heat or cold	0% (0)	0% (0)	44.4% (4)	55.6% (5)	1
Jungle	0% (0)	11.1% (1)	44.4% (4)	44.4% (4)	0.89
Cave exploration	0% (0)	0% (0)	66.7% (6)	33.3% (3)	1
Hiking or trekking	0% (0)	11.1% (1)	66.7% (6)	22.2% (2)	0.89
Rafting or kayaking	0% (0)	22.2% (2)	55.6% (5)	22.2% (2)	0.78
Restricted work camp	0% (0)	22.2% (2)	55.6% (5)	22.2% (2)	0.78
Motorcycle	0% (0)	11.1% (1)	33.3% (3)	55.6% (5)	0.89
Backpacking	0% (0)	22.2% (2)	33.3% (3)	44.4% (4)	0.78
Trekking	0% (0)	33.3% (3)	33.3% (3)	33.3% (3)	0.67
Friend/family cooking	0% (0)	0% (0)	22.2% (2)	77.8% (7)	1
Street food and vendors	0% (0)	0% (0)	33.3% (3)	66.7% (6)	1
Ave-CVI = 0.91					

**Table 4:** Pharmacist participant characteristics.

Characteristic	Frequency (%) (n = 8)
**Type of community pharmacy**	
Chain	4 (50.0%)
Independent	1 (12.5%)
Banner	3 (37.5%)
**Role in pharmacy**	
Staff pharmacist	4 (50.0%)
Owner	2 (25.0%)
Pharmacy student	2 (25.0%)
Designated manager^1^	3 (37.5%)
**Location in Ontario**	
Central South	1 (12.5%)
Central West	1 (12.5%)
East	1 (12.5%)
South West	5 (62.5%)
**Years in a community pharmacy practice (licensed pharmacists only, n = 6)**	
Less than 1	1 (16.7%)
11–20	4 (66.6%)
21–30	1 (16.7%)
**Average number of hours worked per week (licensed pharmacists only, n = 6)**	
8–16	2 (33.3%)
25–32	1 (16.7%)
33–40	2 (33.3%)
More than 40	1 (16.7%)
**Gender**	
Male	3 (37.5%)
Female	5 (62.5%)
**Authorized to administer injections**	
Yes	8 (100.0%)
**Education (licensed pharmacists only, n = 6), select all that apply**	
BSc Pharmacy	5 (83.3%)
Entry-to-practice PharmD	1 (16.7%)

^1^ Participants had the option to select designated manager of the pharmacy in addition to other roles.

**Table 5:** Framework metrics collected by pharmacists.

Date Used (YYYY/MM/DD)	Estimated Triage Time (mins)	Did You Refer the Patient?	What Was the Reason for Referring/Not Referring	If You Did Not Refer, What Was the Course of Action?
2019/03/26	45*	Yes	“Needed yellow fever vaccine, proof of polio vaccination and malaria chemoprophylaxis”	
2019/03/28	35*	Yes	“Needed yellow fever vaccine and proof of polio vaccination”	
2019/04/20	15	No	“Did not refer as not high risk”	“Patient had TwinRix^®^ [combined hepatitis A and B vaccine] previously and decided to get Dukoral^®^ [oral cholera vaccine]”

* Interaction was performed by 4th year entry-to-practice PharmD student.

## Appendix A

**Table A1:** Pre-Test Survey Questions.

Question	Answer Options
**Screening**	
Do you currently work in a community pharmacy practice setting?	Yes No
Do you currently have an Ontario Part A license to practice pharmacy in the province?	Yes No
Do you currently have the Certificate in Travel Health^TM^ from the International Society of Travel Medicine?	Yes No
**Demographics**	
Which type of community pharmacy practice setting do you primarily work in?	Independent community pharmacy Community pharmacy associated with a chain Community pharmacy associated with a banner Community pharmacy associated with a grocery store Community pharmacy associated with a mass merchandiser Other (please specify)
What is your role in the community pharmacy practice setting you work in?	Community pharmacy owner Community pharmacy staff pharmacist Community pharmacy relief pharmacist
Are you the pharmacy’s designated manager?	Yes No
Where is your community pharmacy practice setting located?	Central East Central South Central West East North South West Toronto
How many years have you worked in a community pharmacy practice setting?	Currently on a community pharmacy rotation Less than 1 1-5 6-10 11-20 21-30 More than 30
On average, how many hours per week do you work in a community pharmacy practice setting?	Currently on a community pharmacy rotation Less than 8 8-16 17-24 25-32 33-40 More than 40
Which gender do you most identify with?	Male Female Gender variant/non-conforming
Are you authorized to administer injections in Ontario?	Yes No
What degrees/training have you received? Select all that apply.	Currently on clinical rotations for entry-to-practice PharmD BSc Pharmacy Post-baccalaureate PharmD Entry-to-practice PharmD Masters in Pharmacy PhD in Pharmacy Residency Fellowship Other (please specify)
Which of the following travel or travel-related vaccines have you personally administered since the expansion of Ontario pharmacists’ scope in December 2016? Select all that apply.	Bacille Calmette-Guerin (BCG) (for tuberculosis) Haemophilus influenza type B Hepatitis A Hepatitis B Combined hepatitis A and B Herpes zoster (shingles) Human papillomavirus (HPV) Japanese encephalitis Meningitis Pneumococcus Rabies Typhoid Combined typhoid and hepatitis A Varicella zoster (chickenpox) Yellow Fever None of the above
Does your pharmacy currently offer travel health services other than administration of travel vaccines (e.g., pretravel consultations)?	Yes No
**Pharmacy Practice**	
What do you do when a patient presents to the pharmacy wondering what precautions they need for upcoming their travel destination? Select all that apply.	Refer all patients to a travel clinic or to their physician Provide information on general precautions Refer them to online or paper resources Perform a complete consultation for less complex patients only (e.g., all-inclusive resort in the Caribbean, cruise) and refer all others Other (please specify)
Please describe your current willingness to incorporate travel medicine services at your pharmacy.	Free-text response
Please describe the primary barrier(s) preventing your pharmacy from starting travel medicine services	Free-text response
Please describe the primary motivator(s) for your pharmacy wanting to start travel medicine services	Free-text response

**Table A2:** Post-Test Survey Questions.

Question	Answer Options
**Practice Questions**	
What do you do when a patient presents to the pharmacy wondering what precautions they need for upcoming their travel destination? Select all that apply.	Refer all patients to a travel clinic or to their physician Provide information on general precautions Refer them to online or paper resources Perform a complete consultation for less complex patients only (e.g., all-inclusive resort in the Caribbean, cruise) and refer all others Other (please specify)
Please describe your current willingness to incorporate travel medicine services at your pharmacy.	Free-text response
Please describe the primary barrier(s) preventing your pharmacy from starting travel medicine services.	Free-text response
Please describe the primary motivator(s) for your pharmacy wanting to start travel medicine services.	Free-text response
When and how do you currently offer travel consultations at your pharmacy? Select all that apply.	Anytime by walk-in During set days/hours by walk-in (e.g., clinic days) By appointment Other (please specify)
Does your pharmacy charge a fee to patients for a travel consultation?	Yes (please specify the fee amount) No
**Framework**	
Please describe the main advantages of the framework.	Free-text response
Please describe the main disadvantages of the framework.	Free-text response
Please provide any suggestions, improvements, or clarifications needed for future editions of the framework	Free-text response

## Appendix B

**Table A3:** Summary of Item Results from Content Judgement Phase.

**Domain: Who?**	**Essential** **(%, n)**	**Useful, but Not Essential** **(%, n)**	**Not Necessary** **(%, n)**
**Health Conditions**			
Diabetes*	66.7% (6)	11.1% (1)	0% (0)
High blood pressure	11.1% (1)	55.6% (5)	11.1% (1)
High cholesterol	0% (0)	66.7% (6)	11.1% (1)
Blood or clotting disorder*	77.8% (7)	0% (0)	0% (0)
Heart disease or arrhythmia*	66.7% (6)	11.1% (1)	0% (0)
Seizure disorder*	66.7% (6)	11.1% (1)	0% (0)
Emotional/psychiatric condition(s) *	77.8% (7)	0% (0)	0% (0)
Lung condition (Asthma/COPD)	44.4% (4)	33.3% (3)	0% (0)
Migraines or headaches	0% (0)	66.7% (6)	11.1% (1)
Irritable Bowel Syndrome or digestive tract problems	22.2% (2)	44.4% (4)	11.1% (1)
Inflammatory Bowel Disease*	55.6% (5)	22.2% (2)	0% (0)
Acid Reflux or heartburn	33.3% (3)	44.4% (4)	0% (0)
Thymus disorders (e.g., myasthenia gravis) *	66.7% (6)	11.1% (1)	0% (0)
Radical mastectomy or lymph-node dissection	44.4% (4)	33.3% (3)	0% (0)
Liver or kidney disease*	77.8% (7)	0% (0)	0% (0)
Damaged or removed spleen*	77.8% (7)	0% (0)	0% (0)
Organ or bone marrow transplant*	77.8% (7)	0% (0)	0% (0)
Recent chemotherapy or radiation (4 months) *	77.8% (7)	0% (0)	0% (0)
HIV, AIDS, immune suppressed or immunocompromised*	77.8% (7)	0% (0)	0% (0)
Psoriasis	44.4% (4)	33.3% (3)	0% (0)
Ear/hearing problems	0% (0)	77.8% (7)	0% (0)
Anemia	11.1% (1)	66.7% (6)	0% (0)
**Considerations for Females when Traveling**			
Currently pregnant*	77.8% (7)	0% (0)	0% (0)
Planning to get pregnant soon after travel*	77.8% (7)	0% (0)	0% (0)
Breastfeeding*	55.6% (5)	22.2% (2)	0% (0)
Date of last menstrual period	22.2% (2)	33.3% (3)	22.2% (2)
**Demographics**			
Age*	77.8% (7)	0% (0)	0% (0)
Date of birth (for immunization purposes) *	55.6% (5)	22.2% (2)	0% (0)
**Medications**			
Blood thinners (e.g., warfarin, clopidogrel) *	77.8% (7)	0% (0)	0% (0)
Corticosteroids*	66.7% (6)	11.1% (1)	0% (0)
Chemotherapy or other anti-cancer medications*	77.8% (7)	0% (0)	0% (0)
Quinine, quinidine, or other cardiac drugs*	66.7% (6)	11.1% (1)	0% (0)
Antibiotics	33.3% (3)	44.4% (4)	0% (0)
Medication for mood disorders or emotional problems*	55.6% (5)	22.2% (2)	0% (0)
Medications to control seizures*	55.6% (5)	22.2% (2)	0% (0)
Other prescription medications	33.3% (3)	44.4% (4)	0% (0)
**Allergy**			
Sulfa drugs	22.2% (2)	55.6% (5)	0% (0)
Streptomycin, gentamicin or neomycin*	55.6% (5)	22.2% (2)	0% (0)
Penicillin	22.2% (2)	55.6% (5)	0% (0)
Latex	44.4% (4)	33.3% (3)	0% (0)
Yeast	44.4% (4)	33.3% (3)	0% (0)
Gelatin	33.3% (3)	44.4% (4)	0% (0)
Eggs or other foods	44.4% (4)	33.3% (3)	0% (0)
Adhesive bandages	16.7% (1)	83.3% (5)	0% (0)
**Travel Companion**			
Alone	33.3% (3)	44.4% (4)	0% (0)
With spouse/partner	0% (0)	66.7% (6)	11.1% (1)
With a group	0% (0)	66.7% (6)	11.1% (1)
With children*	55.6% (5)	22.2% (2)	0% (0)
With an older/elderly person	57.4% (4)	33.3% (3)	0% (0)
**Immunization History**			
In what country were you born?	44.4% (4)	33.3% (3)	0% (0)
If you were not born in Canada, at what age did you leave your country of birth?†	55.6% (5)	22.2% (2)	0% (0)
Determining if the patient is aware of their immunization status*	77.8% (7)	0% (0)	0% (0)
Has fainted or felt unwell after an injection	33.3% (3)	33.3% (3)	11.1% (1)
Had a serious reaction in the past with vaccines*	77.8% (7)	0% (0)	0% (0)
Had (or currently has) a fear of needles	33.3% (3)	44.4% (4)	0% (0)
Carries an Epi-Pen	33.3% (3)	33.3% (3)	11.1% (1)
**Domain: Where?**	**Essential** **(%, n)**	**Useful, but not essential** **(%, n)**	**Not necessary** **(%, n)**
**Destination(s)**			
Country/Countries*	77.8% (7)	0% (0)	0% (0)
Cities/Regions*	66.7% (6)	11.1% (1)	0% (0)
Dates of travel for each country and/or city (if more than one)*	77.8% (7)	0% (0)	0% (0)
Rural/urban areas*	77.8% (7)	0% (0)	0% (0)
**Accommodations**			
Premium hotel	44.4% (4)	22.2% (2)	11.1% (1)
Budget hotel	44.4% (4)	33.3% (3)	0% (0)
Resort	33.3% (3)	44.4% (4)	0% (0)
Cruise	44.4% (4)	33.3% (3)	0% (0)
Hostel*	55.6% (5)	22.2% (2)	0% (0)
Friends/family’s home*	77.8% (7)	0% (0)	0% (0)
Camping*	55.6% (5)	22.2% (2)	0% (0)
**Dining**			
Local restaurants/bars	33.3% (3)	44.4% (4)	0% (0)
Cooking themselves	33.3% (3)	44.4% (4)	0% (0)
Friend/family cooking*	55.6% (5)	22.2% (2)	0% (0)
Street food and vendors*	55.6% (5)	22.2% (2)	0% (0)
**Domain: When?**	**Essential** **(%, n)**	**Useful, but not essential** **(%, n)**	**Not necessary** **(%, n)**
**Timing**			
Departure/Arrival Dates*	55.6% (5)	22.2% (2)	0% (0)
Last minute traveler (<4 weeks before departure date)*	77.8% (7)	0% (0)	0% (0)
Time of year	44.4% (4)	33.3% (3)	0% (0)
Length of stay*	66.7% (6)	11.1% (1)	0% (0)
**Domain: Why?**	**Essential** **(%, n)**	**Useful, but not essential** **(%, n)**	**Not necessary** **(%, n)**
**Reason(s) for Travel**			
Visiting friends/family*	77.8% (7)	0% (0)	0% (0)
Business	33.3% (3)	44.4% (4)	0% (0)
Athletic competition*	55.6% (5)	22.2% (2)	0% (0)
Religion (e.g., Hajj)*	66.7% (6)	11.1% (1)	0% (0)
Medical tourism*	77.8% (7)	0% (0)	0% (0)
Sexual tourism*	77.8% (7)	0% (0)	0% (0)
Humanitarian work*	77.8% (7)	0% (0)	0% (0)
Vacation	44.4% (4)	33.3% (3)	0% (0)
Adventure*	66.7% (6)	11.1% (1)	0% (0)
Research/Education*	55.6% (5)	22.2% (2)	0% (0)
Adoption*	55.6% (5)	22.2% (2)	0% (0)
**Domain: What?**	**Essential** **(%, n)**	**Useful, but not essential** **(%, n)**	**Not necessary** **(%, n)**
**Planned Activities**			
Scuba diving*	66.7% (6)	11.1% (1)	0% (0)
Going to high altitude*	77.8% (7)	0% (0)	0% (0)
Safari*	55.6% (5)	22.2% (2)	0% (0)
Spending time in rural communities or remote areas*	66.7% (6)	11.1% (1)	0% (0)
Adventure travel*	55.6% (5)	22.2% (2)	0% (0)
Close contact with animals*	77.8% (7)	0% (0)	0% (0)
Providing medical care*	77.8% (7)	0% (0)	0% (0)
Exposure to extreme heat or cold*	66.7% (6)	11.1% (1)	0% (0)
Jungle*	66.7% (6)	11.1% (1)	0% (0)
Cave exploration*	66.7% (6)	11.1% (1)	0% (0)
Hiking or trekking*	66.7% (6)	11.1% (1)	0% (0)
Rafting or kayaking*	66.7% (6)	11.1% (1)	0% (0)
Restricted work camp*	66.7% (6)	11.1% (1)	0% (0)
Misc. excursion off resort	44.4% (4)	33.3% (3)	0% (0)
**Transportation**			
Train	11.1% (1)	66.7% (6)	0% (0)
Rental car	22.2% (2)	55.6% (5)	0% (0)
In-country flights	11.1% (1)	66.7% (6)	0% (0)
Boat	44.4% (4)	33.3% (3)	0% (0)
Motorcycle*	55.6% (5)	22.2% (2)	0% (0)
**Type of Travel**			
Package	22.2% (2)	44.4% (4)	11.1% (1)
Camping	33.3% (3)	44.4% (4)	0% (0)
Self-organized	22.2% (2)	44.4% (4)	11.1% (1)
Cruise ship	33.3% (3)	44.4% (4)	0% (0)
Backpacking*	55.6% (5)	22.2% (2)	0% (0)
Trekking*	55.6% (5)	22.2% (2)	0% (0)
**Domain: How?**	**Essential** **(%, n)**	**Useful, but not essential** **(%, n)**	**Not necessary** **(%, n)**
**Travel Experience**			
New traveler	33.3% (3)	44.4% (4)	0% (0)
Local trips only, never overseas	22.2% (2)	55.6% (5)	0% (0)
Travelled overseas	22.2% (2)	55.6% (5)	0% (0)
Experienced traveler	22.2% (2)	55.6% (5)	0% (0)

* Item considered ‘essential’ and included in stage 3. † Not included in stage 3, as it was a follow-up question to “In what country were you born?”.
